# Supplementary material for: Exploring tissue morphodynamics using the photoconvertible Kaede protein in amphioxus embryos
Source: PLoS One. 2022 Sep 27;17(9):e0275193. doi: 10.1371/journal.pone.0275193 (PMC9514637; doi:10.1371/journal.pone.0275193)
Supplement: S1 File — (PDF) [file pone.0275193.s001.pdf]

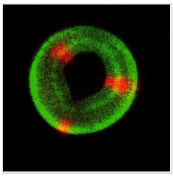

Sep 08, 2022

Exploring tissue morphodynamics using the photoconvertible Kaede protein in amphioxus embryos

Lydvina Meister[1], Hector Escriva[1], Stephanie Bertrand[1]

[1][Sorbonne Université, CNRS, Biologie Intégrative des Organismes Marins, BIOM, F-66650, Banyuls-sur-Mer, France]

2 Works for me Share

dx.doi.org/10.17504/protocols.io.j8nlk46z6g5r/v1

Stephanie Bertrand

ABSTRACT

Photoconvertible proteins are powerful tools widely used in cellular biology to study cell dynamics and organelles. Over the past decade, photoconvertible proteins have also been used for developmental biology applications to analyze cell lineage and cell fate during embryonic development. One of these photoconvertible proteins called Kaede, from the stony coral *Trachyphyllia geoffroyi,* undergoes irreversible photoconversion from green to red fluorescence when illuminated with UV light. Undertaking a cell tracing approach using photoconvertible proteins can be challenging when using unconventional animal models. In this protocol, we describe the use of Kaede to track specific cells during embryogenesis of the cephalochordate *Branchiostoma lanceolatum*. This protocol can be adapted to other unconventional models, especially marine animals.

DOI

dx.doi.org/10.17504/protocols.io.j8nlk46z6g5r/v1

PROTOCOL CITATION

Lydvina Meister, Hector Escriva, Stephanie Bertrand 2022. Exploring tissue morphodynamics using the photoconvertible Kaede protein in amphioxus embryos. **protocols.io**
https://protocols.io/view/exploring-tissue-morphodynamics-using-the-photocon-buj5nuq6

FUNDERS ACKNOWLEDGEMENT

Agence Nationale de la Recherche
Grant ID: ANR-19-CE13-0011-01

Agence Nationale de la Recherche
Grant ID: ANR-16-CE12-0008-01

H2020-INFRAIA-1-2016- 2017
Grant ID: 730984

KEYWORDS

Kaede, photoconversion, development, cell tracing, amphioxus, cephalochordate, embryo, fluorescent protein, confocal, FRAP wizard

LICENSE

This is an open access protocol distributed under the terms of the Creative Commons Attribution License, which permits unrestricted use, distribution, and reproduction in any medium, provided the original author and source are credited

Citation: Lydvina Meister, Hector Escriva, Stephanie Bertrand Exploring tissue morphodynamics using the photoconvertible Kaede protein in amphioxus embryos https://dx.doi.org/10.17504/protocols.io.j8nlk46z6g5r/v1

This is an open access protocol distributed under the terms of the **Creative Commons Attribution License** (https://creativecommons.org/licenses/by/4.0/), which permits unrestricted use, distribution, and reproduction in any medium,

CREATED

Apr 28, 2021

LAST MODIFIED

Sep 08, 2022

PROTOCOL INTEGER ID

49501

1 **Preparation of the Kaede mRNA**

1.1 2h 30m

Plasmid linearization

1. Mix together:

- **5 µg** of the pCS2+ plasmid containing the coding sequence of the Kaede protein from *Trachyphyllia geoffroyi* (AB085641.1)
- **5 µL** of NotI - 500 units **New England Biolabs Catalog #R0189S**
- **20 µL** of CutSmart® Buffer **New England Biolabs Catalog #B7204S**
- Nuclease-free Water **Contributed by users** q.s. **200 µL**

2. Incubate **02:00:00** at **37 °C** .

3. Add:

- **20 µL** of ammonium acetate **Contributed by users** [M]**3 Molarity (M)**
- **400 µL** of Ethanol **Contributed by users**

4. Incubate at **-20 °C** **Overnight** .

5. Centrifuge **13000 rpm, 4°C, 00:20:00** .

6. Discard the supernatant.

7. Add **600 µL** of cold Ethanol 70% **Contributed by users** .

8. Centrifuge **13000 rpm, 4°C, 00:10:00** .

9. Discard the supernatant and let the pellet air dry.

10. Disolve the pellet in **10 µL** of Nuclease-free Water **Contributed by users** .

11. Run **0.5 µL** in an [M]**1 Mass / % volume** Agarose **Sigma Aldrich Catalog #A9539** gel to test for complete linearization.

12. Measure the plasmid concentration using **0.5 µL** with a

Nanodrop 2000C
Thermo Scientific TSC-ND2000C

or equivalent equipment.

Citation: Lydvina Meister, Hector Escriva, Stephanie Bertrand Exploring tissue morphodynamics using the photoconvertible Kaede protein in amphioxus embryos https://dx.doi.org/10.17504/protocols.io.j8nlk46z6g5r/v1

This is an open access protocol distributed under the terms of the **Creative Commons Attribution License** (https://creativecommons.org/licenses/by/4.0/), which permits unrestricted use, distribution, and reproduction in any medium,

1.2 mRNA synthesis

3h 15m

Use the mMESSAGE mMACHINE™ SP6 Transcription Kit **Thermo Fisher Catalog #AM1340** and follow the manufacturer's instructions.

1. Thaw the frozen reagents.
2. Mix together:
   - **10 µL** of 2X NTP/CAP
   - **2 µL** of 10X Reaction Buffer
   - **1 µg** of the linearized plasmid
   - **2 µL** of Enzyme mix
   - Nuclease-free Water **Contributed by users** q.s. **20 µL**
3. Incubate at **37 °C** during **02:00:00** .
4. Add **1 µL** TURBO DNase and incubate at **37 °C** during **00:15:00** .
5. Add **30 µL** of Nuclease-free Water **Contributed by users** and **30 µL** of LiCl Precipitation Solution.
6. Mix and incubate at **-20 °C** during at least **00:30:00** .
7. Centrifuge **13000 rpm, 4°C, 00:20:00** .
8. Remove the supernatant and add **1 mL** of Ethanol 70% **Contributed by users** .
9. Centrifuge **13000 rpm, 4°C, 00:10:00** .
10. Remove the supernatant.
11. Let the pellet air dry and resuspend in **10 µL** of Nuclease-free Water **Contributed by users** .
12. Mesure the concentration and check for the integrity of the RNA on a [M] **1 Mass / % volume** Agarose **Sigma Aldrich Catalog #A9539** gel.
13. The mRNA is either kept in small aliquots at **-80 °C** for upcoming experiments or directly used for the microinjection experiment.

If the concentration is low (less than [M] **2 µg/µL**), you can undertake several synthesis reactions, precipitate them together and resuspend the pellet in a smaller volume.

2 Gametes obtaining

2.1 Adult collection

1d

Ripe *Branchiostoma lanceolatum* adults are obtained by sieving the sand collected at a 5 m depth near the Racou beach (Argelès sur Mer, France) during the months of May and June.

Citation: Lydvina Meister, Hector Escriva, Stephanie Bertrand Exploring tissue morphodynamics using the photoconvertible Kaede protein in amphioxus embryos https://dx.doi.org/10.17504/protocols.io.j8nlk46z6g5r/v1

This is an open access protocol distributed under the terms of the **Creative Commons Attribution License** (https://creativecommons.org/licenses/by/4.0/), which permits unrestricted use, distribution, and reproduction in any medium,

They are kept in small seawater tanks with few centimeters of sand. The temperature is maintained at 17 °C and a light/dark cycle of 14:00:00 / 10:00:00 is applied. The seawater is changed three times per day.

2.2 Spawning induction

Gametes are obtained using the heat stimulation method published in:

Fuentes M, Benito E, Bertrand S, Paris M, Mignardot A, Godoy L, Jimenez-Delgado S, Oliveri D, Candiani S, Hirsinger E, D'Aniello S, Pascual-Anaya J, Maeso I, Pestarino M, Vernier P, Nicolas JF, Schubert M, Laudet V, Geneviere AM, Albalat R, Garcia Fernandez J, Holland ND, Escriva H (2007). Insights into spawning behavior and development of the European amphioxus (Branchiostoma lanceolatum).. Journal of experimental zoology. Part B, Molecular and developmental evolution.

Amphioxus spawn at night, just after the sunset. Briefly, ripe adults kept at 17 °C are placed in a tank without sand at 23 °C the day before spawning, with the same day/night cycle. Two hours before the light is turned off the spawning day, animals are individualized in plastic cups filled with a small volume of filtered seawater and placed on a black background in order to facilitate the vizualisation of the gametes as they are released. After one hour in the dark, the gametes are collected: the sperm diluted in the seawater is conserved at 4°C and can be used during several hours. The eggs are collected by pipetting and placed in small scratched Petri dishes to avoid sticking to the plastic.

3 Oocytes injection

Microinjection is undertaken following the protocol published previously in:

Hirsinger E, Carvalho JE, Chevalier C, Lutfalla G, Nicolas JF, Peyriéras N, Schubert M (2015). Expression of fluorescent proteins in Branchiostoma lanceolatum by mRNA injection into unfertilized oocytes.. Journal of visualized experiments : JoVE. https://doi.org/10.3791/52042

3.1 Material to prepare in advance

5m

1. Injection needles are prepared using Thin-Wall Capillary 4 1.0mm (fil) **World Precision Instruments Catalog #TW100F-4** capillaries and a micropipette puller as for example:

Citation: Lydvina Meister, Hector Escriva, Stephanie Bertrand Exploring tissue morphodynamics using the photoconvertible Kaede protein in amphioxus embryos https://dx.doi.org/10.17504/protocols.io.j8nlk46z6g5r/v1

This is an open access protocol distributed under the terms of the **Creative Commons Attribution License** (https://creativecommons.org/licenses/by/4.0/), which permits unrestricted use, distribution, and reproduction in any medium,

P-97 micropipette puller
Flaming/Brown Micropipette Puller
Sutter instrument N/A

2. Poly-D-lysine coated dishes are prepared as follows:
- Dilute the POLY-D-LYSINE HYDROBROMIDE MOL WT 70000 - 5MG **Sigma Aldrich Catalog #P6407-5MG** in MilliQ water **Contributed by users** to a final concentration of **0.1 mg/mL** .
- Cover the bottom of Falcon™ Easy-Grip Tissue Culture Dishes **Fisher Scientific Catalog #10038820** with this solution.
- Incubate at **Room temperature** during **00:05:00** .
- Remove the solution.
- Let the dishes air dry.
- Keep the dishes at **4 °C** until use.

3.2 Microinjection

1. Prepare a mix containing as final concentrations:
- **1.5 µg/µL** of Kaede mRNA
- **18 % volume** of Glycerol - for molecular biology, ≥99% **Sigma Aldrich Catalog #G5516**
- **18 % volume** of a **10 mg/mL** solution of Fast Green FCF **Sigma Aldrich Catalog #F7252** diluted in MilliQ water **Contributed by users** .

2. Load the injection needle using Eppendorf™ Microloader™ Pipette Tips **Eppendorf Catalog #5242956003** .
3. Place the needle on a micromanipulator and connect it to the microinjector:

Picospritzer III Intracellular Microinjection Dispense System
Injection system
Picospritzer 052-0500-900
100 psi, 2 channel

Citation: Lydvina Meister, Hector Escriva, Stephanie Bertrand Exploring tissue morphodynamics using the photoconvertible Kaede protein in amphioxus embryos https://dx.doi.org/10.17504/protocols.io.j8nlk46z6g5r/v1

This is an open access protocol distributed under the terms of the **Creative Commons Attribution License** (https://creativecommons.org/licenses/by/4.0/), which permits unrestricted use, distribution, and reproduction in any medium,

4. Deposite a line of oocytes in a Falcon™ Easy-Grip Tissue Culture Dishes **Fisher Scientific Catalog #10038820** dish coated with POLY-D-LYSINE HYDROBROMIDE MOL WT 70000 - 5MG **Sigma Aldrich Catalog #P6407-5MG** and filled with filtered seawater.
5. Cut the end of the needle using fine forceps under the binocular. Use a binocular that allows for 200X magnification.
6. Insert the needle into the oocyte and inject a small volume of injection mix (1/100 to 1/50 of the volume of the oocyte). Depending on the size of the needle after cutting, several injection pulses might be necessary to inject a sufficient volume.
7. When all the oocytes are injected, proceed to fertilization. **10 µL** of diluted sperm are sufficient to fertilize 50 injected oocytes. The embryos are detached from the poly-D-lysine coated dish by delicate water waves pushes after fertilization membrane raising, transferred into a clean Petri dish filled with filtered seawater and kept at **19 °C** .

4 Photoconversion

4.1 Preparing the embryo for the photoconversion

30m

1. **00:30:00** before the photoconversion, observe the embryos under a fluorescent binocular using the green filter.
2. Individualize the fluorescent well-developed embryos into glass bottom culture dishes (35 mm) Nunc™ Glass Bottom Dishes, 12mm **Thermo Fisher Catalog #150680** .
Transfer the embryos using a P200 tip to minimize the risk of damage.
3. Maintain the embryo in the smallest possible volume of filtered seawater. In the center of the Petri dish, a volume of **500 µL** of seawater is sufficient for the embryo survival and photoconversion.
4. Place the embryo in the desired orientation (blastopore view in our example) using a 10 cm steel syringe needle tip under the binocular.
5. Transfer the Petri dish under the confocal inverted microscope for the photoconversion. Here we will describe the procedure using the following microscope:

White Light Laser Confocal Microscope<br>Leica TCS SP8 X<br>Confocal Inverted Microscope	
**Leica**	**NA**

However, any confocal inverted microscope equipped with a UV laser and lasers to image the fluorescence emitted by the Kaede protein can be used. The microscope must also allow scan zoom and ROI scanning in order to effectively target a specific region using this protocol for photoconversion. The UV laser intensity and scan time must be adjusted. If a FRAP module or a

Citation: Lydvina Meister, Hector Escriva, Stephanie Bertrand Exploring tissue morphodynamics using the photoconvertible Kaede protein in amphioxus embryos https://dx.doi.org/10.17504/protocols.io.j8nlk46z6g5r/v1

This is an open access protocol distributed under the terms of the **Creative Commons Attribution License** (https://creativecommons.org/licenses/by/4.0/), which permits unrestricted use, distribution, and reproduction in any medium,

photoconversion/photoactivation module is available on the microscope, it can be used following the manufacturer's instructions.

6. Visualize the embryo under the 20x/NA 0.8 objective by setting the white laser excitation wavelength at 508nm.
7. Scan the embryo all along the z-axis in order to see if the specimen is properly positioned.
8. Activate the FRAP-Wizard (Fluorescent Recovery After Photobleaching) module of the LAS X software platform.

4.2 Photoconversion settings

1m

At the top of the FRAP-wizard interface, several buttons corresponding to the different steps are available : “Overview”, “Set Up”, “Bleach”, “Time course”, “Evaluation”. For photoconversion, the "Evaluation" step is not used.

1. *Step 1: Settings for pre- and post- bleach imaging*

- Click on the "Set Up" button and adjust the parameters for imaging the embryo before and after photoconversion.
- Set up the laser and detectors parameters to excite and detect emission of the Kaede in its two states: before (uncleaved, green, Ex508/Em518) and after (cleaved, red, Ex572/Em582) photoconversion.

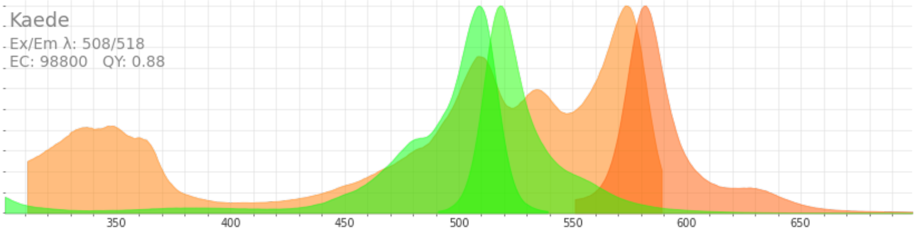

Asset URL:
https://www.fpbase.org/spectra_img/kaede.png?title=1&grid=1

- Adjust the intensity of the laser depending on the amount of Kaede produced by the embryo (correlated to the amount of mRNA injected in the oocyte). Usually, an intensity of 15% is sufficient for imaging the photoconversion.
- Choose the "xyt" acquisition mode for photoconvertion.

2. *Step 2: Parameters for photobleaching*

- Click on the "Bleach" button.
- Set the intensity of the 405nm (UV) diode laser at 85%.
- Turn off the white laser.
- Draw the Regions Of Interest (ROI) on the image of the embryo obtained before the photoconversion. In our example, three circles are drawn, two at the position of the presumptive paraxial mesoderm and one in the putative ventral ectoderm territory.
- Activate the Zoom-In mode (more light is applied to the ROI).
- Turn on the following options: « Set Background to Zero », « Delete Bleach Images after Scan » and « Use laser Settings for all ROIs ».

3. *Step 3: Time Course - Defining the number of prebleach, bleach and postbleach intervals*

- Select the "Time-Course" menu.
- One pre-bleach and one post-bleach intervals are imposed. For photoconversion, use only one

Citation: Lydvina Meister, Hector Escriva, Stephanie Bertrand Exploring tissue morphodynamics using the photoconvertible Kaede protein in amphioxus embryos https://dx.doi.org/10.17504/protocols.io.j8nlk46z6g5r/v1

This is an open access protocol distributed under the terms of the **Creative Commons Attribution License** (https://creativecommons.org/licenses/by/4.0/), which permits unrestricted use, distribution, and reproduction in any medium,

repetition. The photoconversion time depending on the total size of ROIs, adjust the photoconversion session in order to program a total bleaching time of 00:01:00 .

After setting all these parameters, the photobleaching can be started. If the photoconversion is successful, the ROI previously fluorescent after an excitation at 508 nm becomes fluorescent under an excitation at 572 nm. The Petri dish is then filled with 2 mL of filtered seawater and incubated at 19 °C .

5 Imaging embryos at later stages

5m 10s

1. Keep the embryo in the incubator until the desired developmental stage.
2. After 15 hours post fertilization, the embryo starts moving thanks to cilia beating and becomes difficult to scan at high magnification. To immobilise it, incubate the embryo into 2X Artificial Sea Water :

- [M]1038.4 millimolar (mM) Sodium chloride **Sigma – Aldrich Catalog #746398**
- [M]22.2 millimolar (mM) Potassium chloride **Sigma Aldrich Catalog #P9333**
- [M]20 millimolar (mM) Calcium chloride dihydrate **Sigma Aldrich Catalog #C5080**
- [M]49 millimolar (mM) Magnesium chloride hexahydrate **Sigma Aldrich**
- [M]51 millimolar (mM) Magnesium sulfate heptahydrate **Sigma Aldrich Catalog #M2773**

Transfer the specimen with a p20 tip to a Petri dish containing 2X Artificial Sea Water. When the embryo is totally immobilised (around 00:00:10) and sinks to the bottom of the dish, put it back in the initial glass-bottom dish using the same tip. The embryo will remain immobilised for 00:05:00 .

3. Proceed to embryo imaging (z-stack) using excitation at 508 nm (green) and at 572 nm (red) and using the sequential mode. Depending on the developmental stage, the embryo can be imaged on both sides by turning it over delicately after the first scan using a 10 cm steel syringe needle.

Citation: Lydvina Meister, Hector Escriva, Stephanie Bertrand Exploring tissue morphodynamics using the photoconvertible Kaede protein in amphioxus embryos https://dx.doi.org/10.17504/protocols.io.j8nlk46z6g5r/v1

This is an open access protocol distributed under the terms of the **Creative Commons Attribution License** (https://creativecommons.org/licenses/by/4.0/), which permits unrestricted use, distribution, and reproduction in any medium,
