# Supplementary material for: Exploring tissue morphodynamics using the photoconvertible Kaede protein in amphioxus embryos
Source: PLoS One. 2022 Sep 27;17(9):e0275193. doi: 10.1371/journal.pone.0275193 (PMC9514637; doi:10.1371/journal.pone.0275193)
Supplement: Attachment — Submitted filename: Point_by_point_answer.docx [file pone.0275193.s002.docx]

Dear editor,

plesae find below our answers to the comments on our manuscript PONE-D-22-17287, “Exploring tissue morphodynamics using the photoconvertible Kaede protein in amphioxus embryos”. For clarity our answers are in italics and in blue.

**Additional Editor Comments**:

1.File S1, page 4, section 3.2, 1st step; please check the concentration of Fast Green FCF used here (18%). It seems much higher than the listed solubility (1mg/mL) on the Sigma-Aldrich catalog.

*Thank you for pointing out this error. The 18% correspond to the final volume percentage of a solution of fast green diluted in water. The initial solution is at 10mg/mL (the solubility being, contrary to what is indicated in the Sigma-Aldrich catalog, 20g/100ml.Please see:* [*https://pubchem.ncbi.nlm.nih.gov/compound/Fast-Green-FCF#section=Solubility*](https://pubchem.ncbi.nlm.nih.gov/compound/Fast-Green-FCF#section=Solubility)*). We have modified the protocol on protocols.io accordingly.*

2. File S1, page 5, section 3.2, 6th step; it should be more specific on this description of “a small volume”. To my personal knowledge, the right amount of injection volume is crucial for the successful rate of microinjection experiments, thus I would suggest the authors to provide more specific description for determining this injection volume.

*Thank you for the remark. We have modified as follows to be more precise: “Insert the needle into the oocyte and inject a small volume of injection mix (1/100 to 1/50 of the volume of the oocyte). Depending on the size of the needle after cutting, several injection pulses might be necessary to inject a sufficient volume.”*

**Reviewer #1:**

Exploring tissue morphodynamics using the photoconvertible Kaede protein in amphioxus embryos

The protocol is useful for a growing cohort of researchers in this animal. The animal (amphioxus) is not new to research, nor is the protein used (Kaede) but the combination here is quite useful.

*We would like to thank the reviewer for this kind comment.*

“published” protocols.io is not a functional link

*We have now “published” the protocol and give the correct link to the protocols.io file.*

Demonstrable success using this method is given and adequately shows the effectiveness of the protocol and goals.

Has nice links to JOVE references for ease of learning the procedure.

Sufficient detail is given for the protocols, and are comprehensive.

*Thanks again.*

Additional microscope protocols would be helpful since not everyone uses the same as described herein.

*We understand this concern. As requested by PloS ONE for Lab Protocols article, we only described the protocol that has been validated in our laboratory. However, we have added in step 4.1 the following paragraph that should allow researchers having a different microscope to consider undertaking an experiment of this type with their own equipment:*

*“However, any confocal inverted microscope equipped with a UV laser and lasers to image the fluorescence emitted by the Kaede protein can be used. The microscope must also allow scan zoom and ROI scanning in order to effectively target a specific region using this protocol for photoconversion. The UV laser intensity and scan time must be adjusted. If a FRAP module or a photoconversion/photoactivation module is available on the microscope, it can be used following the manufacturer's instructions.”.*

**Reviewer #2:**

The protocol described in this peer-reviewed article (Figure 1) is published already on protocols.io. The method itself is quite straight forward by injecting mRNA of fluorescent protein.

*We are sorry we don’t understand this remark. There is no such protocol in protocols.io (nor on amphioxus injection, nor on Kaede photoconversion).*
